# A57 LONG-TERM EXPOSURE TO ANTICANCER TREATMENTS INCREASES AUTOPHAGY IN ESOPHAGEAL SQUAMOUS CELL CARCINOMA

**DOI:** 10.1093/jcag/gwad061.057

**Published:** 2024-02-14

**Authors:** M Hamilton, J Douchin, B Gaudette, V Giroux

**Affiliations:** Immunologie et Biologie cellulaire, Universite de Sherbrooke, Sherbrooke, QC, Canada; Immunologie et Biologie cellulaire, Universite de Sherbrooke, Sherbrooke, QC, Canada; Immunologie et Biologie cellulaire, Universite de Sherbrooke, Sherbrooke, QC, Canada; Immunologie et Biologie cellulaire, Universite de Sherbrooke, Sherbrooke, QC, Canada

## Abstract

**Background:**

The most common type of esophageal cancer, esophageal squamous cell carcinoma (ESCC), has a 5-year survival rate of only 15%. This low survival rate is to some extent attributed to a high relapse rate, which is, in part, linked to the presence of cancer stem cells (CSC). CSC are a subpopulation of tumor cells that show reduced sensitivity to conventional anticancer therapies, increased potency and self-renewal capacities. With that in mind, ESCC cell lines were exposed in a prolonged manner to anticancer treatments (radiotherapy, 5-FU chemotherapy, and combined therapy) in our laboratory. As expected, an increase in the proportion of CSC (CD44^high^CD24^high^) was observed by flow cytometry following the treatments. In pursuit of innovative therapeutic approaches, we conducted an in-depth investigation of the proteomic profiles of these cell lines by mass spectrometry (MS), seeking to unveil novel biological mechanisms. Interestingly, many proteins associated with metabolism and autophagy were modulated. Several alterations were observed by a metabolomic approach as well, namely an increase in intracellular lactate concentration. Recently, a novel role for lactate has been described: lactylation, which is a post-translational modification. Lactylome of those cell lines revealed alteration in proteins related to autophagy and those results led to my research project.

**Aims:**

Investigate the role of lactylation in autophagy of ESCC.

**Methods:**

Human ESCC cell lines (including the cell lines exposed to anticancer treatments) will be used as cell culture models. Cell and organoid cultures, mass spectrometry, qPCR, WB, and proliferation assay were used.

**Results:**

MS analysis of proteins related to autophagy (i.e. RAB1B, CK2, SNX6 et GABARAP) suggests differential regulation of autophagy in cell lines exposed to prolonged treatments. A strong contribution of the 5-FU treatment to the autophagic modification is observable in the double-treated cells. Thus, we confirmed an increase in the LC3BII/I ratio by WB in the cell lines exposed to anticancer treatments. LC3BII/I WB used in combination with Bafilomycin A1 treatment for inhibition of autophagosome and lysosome fusion is a commonly used read-out for autophagic flux. To further study the link between lactylation, autophagy and CSC, we screened 10 human ESCC cell lines for their LC3BII/I ratio. Four cell lines were selected: TE1, TE2, HCE4 and TE5. Using Bafilomycin A1, we confirmed that the autophagic flux in those cell lines was not blocked. Those cell lines will now be used to perform lactylome analysis of sorted CSC cells.

**Conclusions:**

In brief, our results showed that the autophagic flux is increased in cells exposed to prolonged treatments. This suggests that autophagy could play a role in ESCC response to treatment.

**Funding Agencies:**

CIHRTRIANGLE, Chaires de recherche du Canada, CRCHUS, Université de Sherbrooke

